# Non-invasive measurement of pulse pressure variation using a finger-cuff method in obese patients having laparoscopic bariatric surgery

**DOI:** 10.1007/s10877-020-00614-8

**Published:** 2020-11-10

**Authors:** Moritz Flick, Roman Schumann, Phillip Hoppe, Iwona Bonney, Wilbert Wesselink, Bernd Saugel

**Affiliations:** 1 Department of Anesthesiology, Center of Anesthesiology and Intensive Care Medicine, University Medical Center Hamburg-Eppendorf, Martinistrasse 52, 20246 Hamburg, Germany; 2 Department of Anesthesiology and Perioperative Medicine, Tufts University School of Medicine, Boston, MA USA; 3 Edwards Lifesciences, Irvine, CA USA; 4 Outcomes Research Consortium, Cleveland, OH USA

**Keywords:** Fluid responsiveness, Dynamic preload variable, Clearsight, Nexfin, Hemodynamic monitoring

## Abstract

Pulse pressure variation (PPV) is a dynamic cardiac preload variable used to predict fluid responsiveness. PPV can be measured non-invasively using innovative finger-cuff systems allowing for continuous arterial pressure waveform recording, e.g., the Nexfin system [BMEYE B.V., Amsterdam, The Netherlands; now Clearsight (Edwards Lifesciences, Irvine, CA, USA)] (PPV_Finger_). However, the agreement between PPV_Finger_ and PPV derived from an arterial catheter (PPV_ART_) in obese patients having laparoscopic bariatric surgery is unknown. We compared PPV_Finger_ and PPV_ART_ at 6 time points in 60 obese patients having laparoscopic bariatric surgery in a secondary analysis of a prospective method comparison study. We used Bland–Altman analysis to assess absolute agreement between PPV_Finger_ and PPV_ART_. The predictive agreement for fluid responsiveness between PPV_Finger_ and PPV_ART_ was evaluated across three PPV categories (PPV < 9%, PPV 9–13%, PPV > 13%) as concordance rate of paired measurements and Cohen’s kappa. The overall mean of the differences between PPV_Finger_ and PPV_ART_ was 0.5 ± 4.6% (95%-LoA − 8.6 to 9.6%) and the overall predictive agreement was 72.4% with a Cohen’s kappa of 0.53. The mean of the differences was − 0.7 ± 3.8% (95%-LoA − 8.1 to 6.7%) without pneumoperitoneum in horizontal position and 1.1 ± 4.8% (95%-LoA − 8.4 to 10.5%) during pneumoperitoneum in reverse-Trendelenburg position. The absolute agreement and predictive agreement between PPV_Finger_ and PPV_ART_ are moderate in obese patients having laparoscopic bariatric surgery.

**Electronic supplementary material:**

The online version of this article (10.1007/s10877-020-00614-8) contains supplementary material, which is available to authorized users.

## Introduction

Pulse pressure variation (PPV) is a dynamic cardiac preload variable used to predict fluid responsiveness [1–3]. PPV results from intermittent changes in venous return and cardiac preload during mechanical ventilation [1, 2]. PPV reliably predicts fluid responsiveness in patients with sinus rhythm and controlled mechanical ventilation with a tidal volume of at least 8 mL/kg predicted body weight [4]. PPV is calculated based on the arterial pressure (AP) waveform recorded over several respiratory cycles, usually using an arterial catheter [5].

PPV may also be measured non-invasively using innovative finger-cuff systems allowing for continuous AP waveform recording [6, 7], e.g., with the Nexfin system [BMEYE B.V., Amsterdam, The Netherlands; now Clearsight (Edwards Lifesciences, Irvine, CA, USA)]. Nexfin-derived AP and PPV (PPV_Finger_) have been tested and validated in patients having cardiothoracic [8–10] and major abdominal [11] surgery. Nexfin-derived AP measurements also showed good absolute and trending agreement with invasive AP measurements in obese patients having laparoscopic bariatric surgery [12]. However, the agreement between PPV_Finger_ and PPV derived from an arterial catheter (PPV_ART_) in obese patients having laparoscopic bariatric surgery is unknown.

Therefore, we sought to investigate the absolute and predictive agreement between PPV_Finger_ and PPV_ART_ in obese patients having laparoscopic bariatric surgery. As a secondary endpoint we examined pneumoperitoneum- and reverse Trendelenburg position-induced changes in PPV_ART_.

## Methods

### Study design and patients

This study is a secondary analysis of a prospective method comparison study that investigated continuous non-invasive finger-cuff AP measurements and continuous invasive AP measurements in bariatric surgical patients. The primary prospective study was approved by the Ethics Committee (No. 9743) and patients provided written informed consent. The results from the primary study will be reported separately. In this secondary analysis, we compared PPV_Finger_ and PPV_ART_. This secondary analysis was independently approved by the Tufts Health Sciences Institutional Review Board (No. 11704).

The primary study included adults scheduled for elective laparoscopic bariatric surgery (gastric bypass, sleeve gastrectomy, and gastric banding) with a body mass index (BMI) ≥ 40 kg/m^2^ and American Society of Anesthesiologists physical status classification of < IV. Patients with upper or lower extremity edema, history of ipsilateral axillary or inguinal lymph node dissection, vascular or anatomical abnormalities, carpal tunnel syndrome, negative modified Allen’s test, absence of a palpable ipsilateral ulnar pulse, and atrial fibrillation were excluded. For this secondary analysis, we only included patients with recorded PPV_Finger_ and PPV_ART_ measurements.

### Anesthesia management

General anesthesia was induced with fentanyl, propofol, lidocaine, and rocuronium or succinylcholine. Either sevoflurane or desflurane in combination with fentanyl and hydromorphone were administered for maintenance of general anesthesia. Patients were positioned horizontally during surgical preparation of the abdomen and skin closure. Pneumoperitoneum was established with a target pressure of 15 mmHg. During the laparoscopic surgical procedure all patients were positioned in a reverse-Trendelenburg position. Both arms were positioned and secured on padded arm boards.

### Study measurements

We recorded the AP waveform non-invasively using the Nexfin system with the finger-cuffs placed on the middle phalanx of the middle or ring finger ipsilateral to the radial arterial catheter. The heart reference system was leveled to the right atrium of the patient according to the manufacturer’s specifications. The Nexfin system automatically calculates PPV_Finger_ based on proprietary pulse wave analysis of the non-invasively recorded AP waveform. PPV_Finger_ is calculated every 5 s using a 15 s episode for PPV analysis. The displayed PPV_Finger_ is a 1-min moving average. PPV_Finger_ was the test method.

Simultaneously, we recorded the AP waveform invasively using a 20 gauge radial arterial catheter. The arterial catheter was leveled and zeroed to the right atrium of the patient using a disposable transducer [13]. The AP waveform was tested for its damping properties with a fast-flush test to ensure a high-quality AP signal. PPV_ART_ was automatically calculated using the algorithm of the Philips Intellivue MP 90 patient monitor (Philips Healthcare, Andover, MA, USA), which averages four consecutive 8 s PPV measurements [14–16]. PPV_ART_ was the reference method.

We recorded PPV_Finger_ and PPV_ART_ at 6 predefined time points (T1: within 15 min prior to abdominal insufflation; T2: 3 min after pneumoperitoneum insufflation; T3: 15 min after pneumoperitoneum insufflation; T4: 30 min after pneumoperitoneum insufflation; T5: 45 min after pneumoperitoneum insufflation; T6: 3 min after pneumoperitoneum desufflation). Measurements at T1 and T6 were performed in horizontal position and measurements at T2–5 were performed in 30° reverse-Trendelenburg position. We visually inspected the AP waveform just before each study measurement to exclude AP waveform artifacts or abnormalities.

### Statistical analysis

Descriptive data are shown as median (range) for continuous data and as absolute frequencies and percentages for categorical data. The mean ± standard deviation (SD) was calculated for PPV_Finger_ and PPV_ART_; additionally, we calculated the mean ± SD for PPV_Finger_ and PPV_ART_ separately for episodes with and without pneumoperitoneum. The absolute agreement between PPV_Finger_ and PPV_ART_ was investigated using Bland–Altman analysis accounting for repeated measurements within individuals [17, 18]. The mean of the differences, the SD of the mean of the differences, and the 95% limits of agreement (95%-LoA; i.e., mean of the differences ± 1.96 SD of the mean of the differences) are reported to describe the trueness and precision of agreement [19, 20].

The predictive agreement between PPV_Finger_ and PPV_ART_ for fluid responsiveness was evaluated across previously defined categories reflecting clinical practice using the “gray-zone” approach (PPV < 9%, PPV 9–13%, PPV > 13%) [21, 22]. We separately evaluated the predictive agreement during pneumoperitoneum with adapted PPV categories (PPV < 7%, PPV 7–20%, PPV > 20%) [23]. The concordance rate of paired measurements, defined as the number of concordantly paired measurements divided by the total number of paired measurements, and Cohen’s kappa were calculated to evaluate predictive agreement. We defined a Cohen’s kappa value of < 0 as no agreement, 0–0.20 as slight, 0.21–0.40 as fair, 0.41–0.60 as moderate, 0.61–0.80 as substantial, and 0.81–1.00 as near perfect agreement [24].

To illustrate the impact of pneumoperitoneum and reverse-Trendelenburg positioning on PPV_ART_, we created box plots of all PPV measurements, PPV measurements without pneumoperitoneum, and PPV measurements during pneumoperitoneum. We compared consecutive PPV_ART_ measurements before and after pneumoperitoneum insufflation (T1 vs. T2) and before and after pneumoperitoneum desufflation (T5 vs. T6) using Wilcoxon rank sum test. A p-value less than 0.05 was considered statistically significant.

For statistical analysis, we used Excel (Microsoft, Redmond, Washington, USA), and MedCalc (Version 19.2.0, MedCalc Software Ltd., Ostend, Belgium).

## Results

**Fig. 1:**
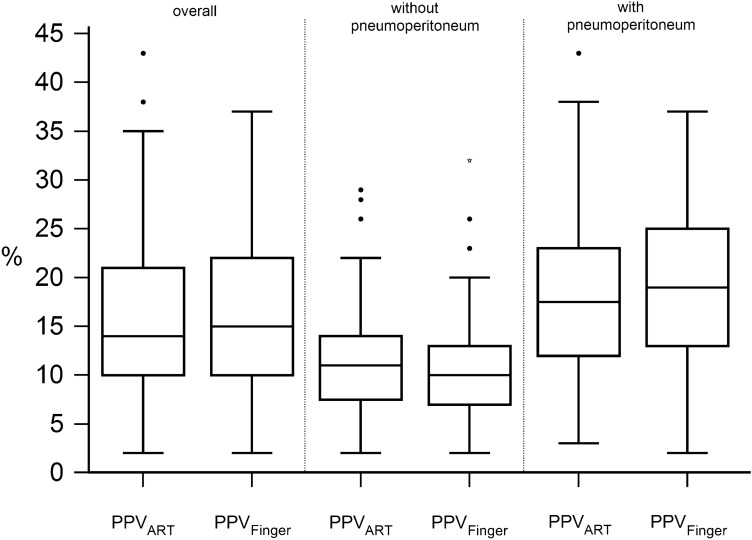
Box plots showing pulse pressure variation (PPV) (%) from the non-invasive finger cuff system Nexfin (PPV_Finger_) and the invasive arterial catheter (PPV_ART_). PPV values are shown as box plots separately for all measurements, measurements without pneumoperitoneum in horizontal position, and measurements during pneumoperitoneum in reverse-Trendelenburg position

We analyzed a total of 337 paired PPV_Finger_ and PPV_ART_ measurements from 60 patients with sinus rhythm. Patient characteristics and procedural data are presented in Table 1. 108 Paired PPV_Finger_ and PPV_ART_ measurements were obtained without pneumoperitoneum in horizontal position, and 229 during pneumoperitoneum in reverse-Trendelenburg position (Fig. 1; Table S1).

**Table 1:** Patient characteristics

Demographic and biometric data	
Male sex [n (%)]	18 (30)
Age (years)	45 (22–72)
Height (cm)	165 (151–198)
Weight (kg)	130 (95–237)
Body mass index (kg/m^2^)	48 (40–70)
American Society of Anesthesiologists Physical Status Class III [n (%)]	60 (100)
Type of surgery	
Laparoscopic sleeve gastrectomy [n (%)]	32 (53)
Laparoscopic gastric bypass [n (%)]	28 (47)
Procedural data	
Duration of anesthesia (h)	3.2 (1.8–5.2)
Administered fluids (mL)	2200 (1200–4200)
Tidal volume (mL/kg predicted body weight) (n = 59)	10.9 (7.5–15.2)
Peak inspiratory pressure (cmH_2_O) (n = 54)	32 (25–40)
Highest positive end-expiratory pressure (cmH_2_O) (n = 31)	5 (4–10)

Data are shown as median (range) or absolute (relative frequencies)

**Fig. 2:**
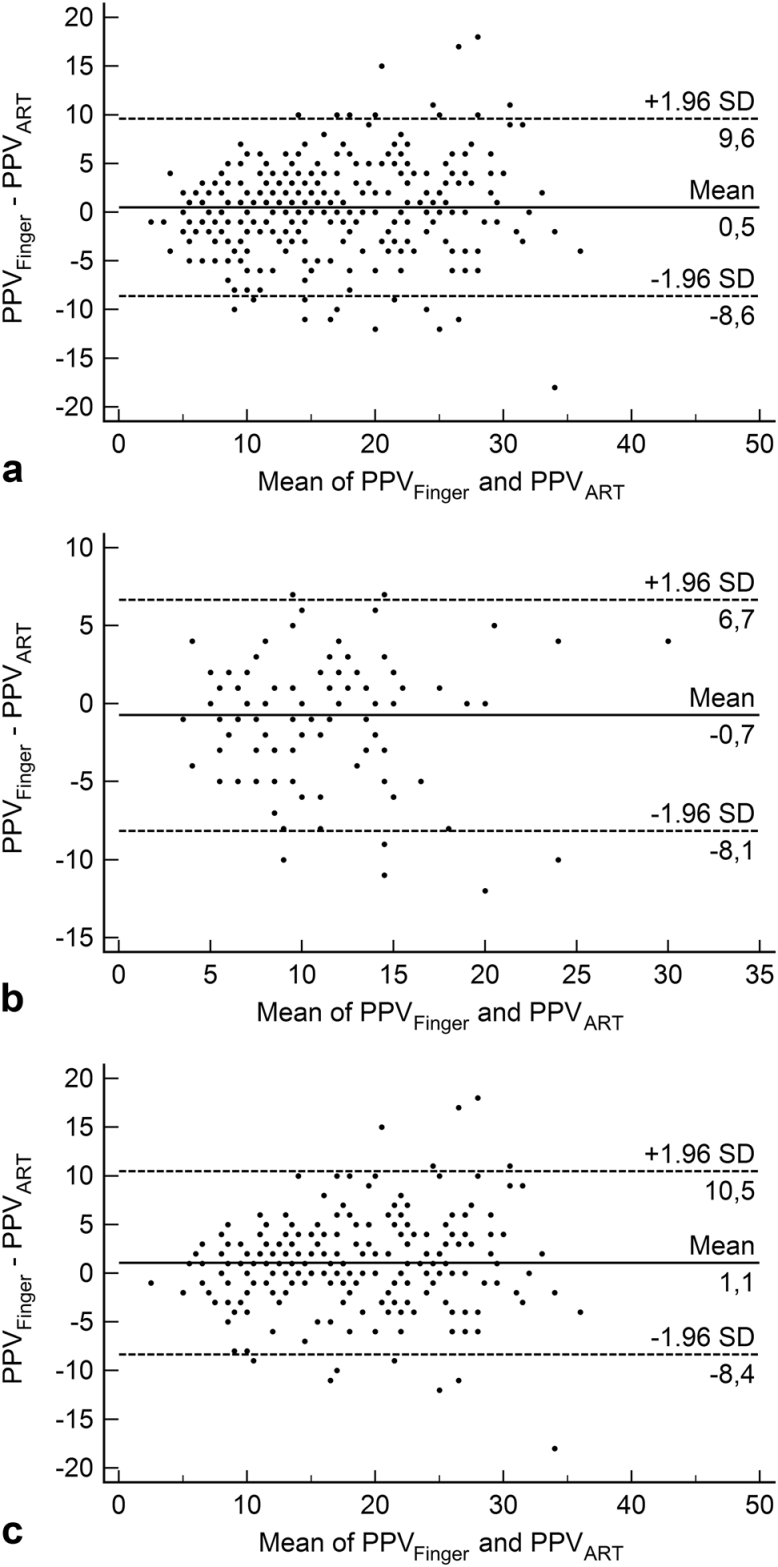
Bland–Altman plots comparing pulse pressure variation (%) from the non-invasive finger cuff system Nexfin (PPV_Finger_) and the invasive arterial catheter (PPV_ART_) for all measurements (**a**), measurements without pneumoperitoneum in horizontal position (**b**), and measurements during pneumoperitoneum in reverse-Trendelenburg position (**c**)

The overall mean of the differences between PPV_Finger_ and PPV_ART_ was 0.5 ± 4.6% (95%-LoA: − 8.6 to 9.6%; Fig. 2a). For measurements without pneumoperitoneum and in horizontal position, the mean of the differences between PPV_Finger_ and PPV_ART_ was − 0.7 ± 3.8% (95%-LoA: − 8.1 to 6.7%; Fig. 2b). During pneumoperitoneum and reverse-Trendelenburg position, the mean of the differences between PPV_Finger_ and PPV_ART_ was 1.1 ± 4.8% (95%-LoA: − 8.4 to 10.5%; Fig. 2c).

The overall predictive agreement between PPV_Finger_ and PPV_ART_ across the predefined categories for fluid responsiveness was 72.4% with a Cohen’s kappa of 0.53 (Table 2). The predictive agreement between PPV_Finger_ and PPV_ART_ without and during pneumoperitoneum is shown in Supplementary Tables S2 and S3.

**Table 2:** Distribution and predictive agreement of pulse pressure variation measurements across the three predefined categories

PPV_ART_	PPV_Finger_			
	< 9%	9–13%	> 13%	
< 9%	38	12	1	
9–13%	22	47	33	Accordance rate : 72.4%
> 13%	*5*	20	159	Cohen’s kappa : 0.53

*PPV*_Finger_ pulse pressure variation measured with Nexfin, *PPV*_ART_ pulse pressure variation measured with the invasive arterial catheter

**Fig. 3:**
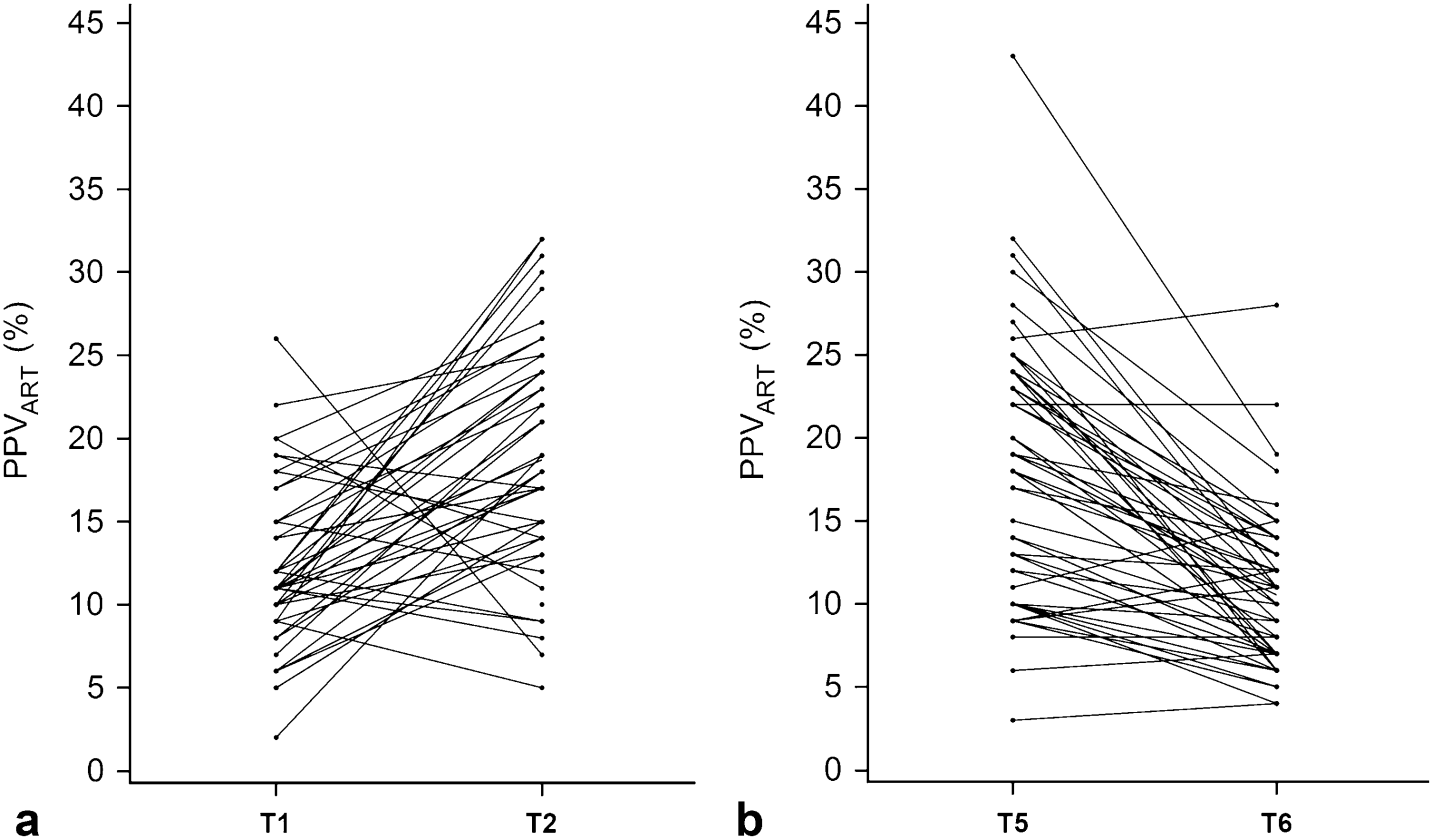
Spaghetti plots illustrating arterial catheter-derived pulse pressure variation (PPV_ART_) (%) before (T1) and after (T2) pneumoperitoneum insufflation and reverse-Trendelenburg positioning (**a**; n = 48) as well as before (T5) and after (T6) pneumoperitoneum desufflation and re-positioning in horizontal position (**b**; n = 56) (both p < 0.0001)

Mean ± SD PPV_ART_ increased from 12.4 ± 5.4% before (T1) to 18.8 ± 6.7% (p < 0.0001) after (T2) pneumoperitoneum insufflation and reverse-Trendelenburg positioning. Pneumoperitoneum desufflation and re-positioning in horizontal position decreased mean PPV_ART_ from 18.3 ± 7.7% (T5) to 10.7 ± 4.6% (T6) (p < 0.0001) (Fig. 3).

## Discussion

We investigated the absolute and predictive agreement between PPV_Finger_ and PPV_ART_ in obese patients having laparoscopic bariatric surgery. The absolute agreement, i.e. the trueness and precision of agreement [19, 20], and the predictive agreement across three predefined PPV categories between PPV_Finger_ and PPV_ART_ were moderate. Pneumoperitoneum insufflation and reverse-Trendelenburg positioning transiently increased PPV_ART_.

Finger-cuff technologies are an alternative to arterial catheters for continuous AP monitoring in morbidly obese patients having surgery [12, 25]. In addition to continuous AP monitoring, finger-cuff technologies allow for calculation of advanced hemodynamic variables including PPV. PPV predicts fluid responsiveness in patients receiving controlled mechanical ventilation and is therefore part of many perioperative goal-directed therapy protocols [4, 26, 27].

In our study, the absolute agreement between PPV_Finger_ and PPV_ART_ was moderate, but worse than in previous studies investigating PPV_Finger_ in non-obese surgical patients. In one of the first studies, the mean of the differences between PPV_Finger_ and PPV_ART_ was − 1.0% (95%-LoA: − 4.3 to 2.4%) in 19 patients after coronary artery bypass graft surgery [10]. Similar results were reported in 19 patients after major abdominal surgery with a mean of the differences of 1.5% (95%-LoA: − 2.7 to 5.7%) [11]. In contrast to these previous studies, we investigated morbidly obese patients having laparoscopic bariatric surgery. Pneumoperitoneum during laparoscopic surgery increases intraabdominal pressure—that is often already elevated at baseline in obese patients—and may thus reduce vascular compliance and venous return resulting in high PPV [28, 29]. Higher overall PPV values in our study may—in part—explain wider 95%-LoA between PPV_Finger_ and PPV_ART_ in the present study compared to previous studies [10, 11]. Additionally, these studies used offline calculation with the same formula for both, PPV_Finger_ and PPV_ART_ [10, 11], whereas we compared PPV_Finger_ automatically calculated using the PPV_Finger_ algorithm and PPV_ART_ calculated by the patient monitor.

We also investigated the predictive agreement between PPV_Finger_ and PPV_ART_ across predefined PPV categories to investigate the ability of PPV_Finger_ to guide fluid therapy. Overall, the predictive agreement was moderate according to Cohen’s kappa for both conventional PPV categories [21] and PPV categories considering pneumoperitoneum [23]. Further studies using fluid challenges to test whether PPV_Finger_ is able to actually predict fluid responsiveness during laparoscopic bariatric surgery in obese patients are needed.

Pneumoperitoneum insufflation increased PPV in our study, which is in agreement with results from experimental studies [30–32] and a study in non-obese patients [33]. Furthermore, positioning patients in reverse-Trendelenburg may have additionally increased PPV because of a further decrease in venous return [34]. The agreement between PPV_Finger_ and PPV_ART_ was slightly worse during pneumoperitoneum and reverse-Trendelenburg positioning compared to without pneumoperitoneum and the horizontal position. However, pneumoperitoneum and reverse-Trendelenburg positioning had little effect on the predictive agreement between PPV_Finger_ and PPV_ART_, which was moderate under both conditions.

Based on our data, the assessment of fluid responsiveness using non-invasive PPV_Finger_ during bariatric surgery should be interpreted cautiously. However, PPV_Finger_ may add an additional measurement contributing to a decision for fluid administration, when additional physiological variables are available.

PPV_ART_ was calculated automatically by the patient monitor. The gold standard for PPV measurement is post hoc manual calculation based on the invasively recorded AP waveform [5]. However, post hoc manual PPV calculation is impractical during routine clinical care. We did not perform any intervention, e.g. fluid challenge, and could thus not evaluate the utility of the Nexfin system to respond to actual fluid administration.

## Conclusions

The absolute agreement and predictive agreement between PPV_Finger_ and PPV_ART_ are moderate in obese patients having laparoscopic bariatric surgery. Pneumoperitoneum and reverse-Trendelenburg positioning transiently increase PPV_ART_.

## Electronic supplementary material

Below is the link to the electronic supplementary material.

(PDF 115 kb)
