# Supplementary material for: Non-invasive measurement of pulse pressure variation using a finger-cuff method in obese patients having laparoscopic bariatric surgery
Source: J Clin Monit Comput. 2020 Nov 10;35(6):1341–7. doi: 10.1007/s10877-020-00614-8 (PMC8542542; doi:10.1007/s10877-020-00614-8)
Supplement: Supplementary file 1 — (PDF 115 kb) [file 10877_2020_614_MOESM1_ESM.pdf]

Table S1 Pulse pressure variation

	Overall (n=337)		Without Pneumoperitoneum (n=108)		With Pneumoperitoneum (n=229)	
Variable	Mean	Standard Deviation	Mean	Standard Deviation	Mean	Standard Deviation
PPV_{ART} (%)	15.7	7.2	11.5	5.0	17.7	7.2
PPV_{Finger} (%)	16.2	7.9	10.6	4.9	18.9	7.6

PPV_{Finger}, pulse pressure variation measured with Nexfin; PPV_{ART}, pulse pressure variation measured with the invasive arterial catheter.

Table S2: Distribution and predictive agreement of pulse pressure variation measurements during pneumoperitoneum across the three predefined categories adapted for laparoscopic surgeries.

		**PPV_{Finger}**			
		<7%	*7 – 20%*	*>20%*	
**PPV_{ART}**	*<7%*	4	5	0	
	7 – 20%	8	100	28	Accordance Rate : 76.0%
	>20%	0	14	70	Cohen's Kappa : 0.55

PPV_{Finger}, pulse pressure variation measured with Nexfin; PPV_{ART}, pulse pressure variation measured with the invasive arterial catheter.

Table S3: Distribution and predictive agreement of pulse pressure variation measurements without pneumoperitoneum across the three predefined categories.

		**PPV_{Finger}**			
		<9%	*9 – 13%*	*>13%*	
**PPV_{ART}**	*<9%*	27	6	0	
	9 – 13%	12	23	9	Accordance Rate : 61.1%
	>13%	3	12	16	Cohen's Kappa : 0.53

PPV_{Finger}, pulse pressure variation measured with Nexfin; PPV_{ART}, pulse pressure variation measured with the invasive arterial catheter.
